# Characterising a Novel Therapeutic Target for Psoriasis, TYK2, Using Functional Genomics

**DOI:** 10.3390/ijms252313229

**Published:** 2024-12-09

**Authors:** Shraddha S. Rane, Sarah Elyoussfi, Elan Shellard, Steve Eyre, Richard B. Warren

**Affiliations:** 1 School of Biological Sciences, Faculty of Biology Medicine and Health, Manchester Academic Health Science Centre, The University of Manchester, Manchester M13 9PT, UK; 2 NIHR Manchester Biomedical Research Centre, Manchester University NHS Foundation Trust, Manchester Academic Health Science Centre, Manchester M13 9PL, UK; 3 Dermatology Centre, Northern Care Alliance NHS Foundation Trust, Manchester M6 8HD, UK

**Keywords:** TYK2, psoriasis, risk allele, protective allele, STAT, deucravacitinib

## Abstract

Psoriasis (Ps) is a debilitating immune-mediated chronic skin condition. It affects about 1–3% of the world population, with an 8–11% prevalence in Northern European populations. Tyrosine kinase 2 (TYK2) is a newly identified target for Ps. An independent non-coding genetic association with Ps has been identified ~400 kb upstream of TYK2. The variants making up the credible Ps Single-Nucleotide Polymorphism (SNP) set were identified in their genomic context with the potential to influence TYK2 expression by interacting with regulatory elements involved in gene regulation. Previous evidence from our laboratory has suggested that credible SNP sets in intronic regions can be distal regulators of the genes of interest through long-range chromatin interactions. We hypothesise that SNPs at ILF3 are distal regulators of TYK2 expression via long-range chromatin interactions and Ps risk. The dysregulation of the TYK2 pathway in Ps may be mediated by a combination of GWAS risk SNPs at ILF3 and TYK2 and downstream genes. We investigated this by employing functional genomics and molecular biology methods. We developed a CD4 T cell model system with Jurkat-dCAS9-VP64 and Jurkat-dCAS9-KRAB cells using CRISPR activation and CRISPR inhibition of the risk variants rs892086 and rs7248205, selected from the latest Ps GWAS SNP set for their long-range interaction and light Linkage Disequilibrium (R^2^ > 0.8), respectively. Using CRISPR activation, we demonstrate here that these risk SNPs, although distal to TYK2, do indeed regulate the TYK2 gene. Investigations into annotating the TYK2 pathway using RNA-seq analysis revealed differentially regulated genes, including VEGFA, C1R, ADORA1, GLUD2, NDUFB8, and FCGR2C, which are thought to be implicated in Ps. These genes were observed to be associated with conditions such as psoriatic arthritis, atopic dermatitis, and systemic sclerosis when compared using published databases, which confirms their relevance and importance in inflammatory conditions. With the developed cell model systems using CRISPR technology and differential gene regulation, we demonstrate here that these genes have the potential to define the TYK2/Ps pathway and our understanding of the disease biology.

## 1. Introduction

Psoriasis (Ps) is a chronic, immune-mediated skin disorder with a strong genetic basis. It affects 2–3% of the global population [1], with an 8–11% prevalence in Northern European populations [2] and an increasing year-on-year incidence [3].

Tyrosine kinase 2 (TYK2) was identified as a Ps risk gene in 2010 [4,5], with its genetic variation associated with several other autoimmune diseases, including multiple sclerosis, systemic sclerosis, Type 1 diabetes, Crohn’s disease, ulcerative colitis, and systemic lupus erythematosus [6]. TYK2 is part of the JAK kinase family, also consisting of JAK1, JAK2, and JAK3, all of which are involved in intracellular signalling post cytokine stimulation. TYK2 is specifically involved in Ps pathogenesis, mediating intracellular signalling for both adaptive and innate immune responses post stimulation with IL-23/IL-12 and type 1 interferons (IFNs), respectively [7]. The IL-23/IL-17 axis in particular is a key signalling pathway in Ps pathogenesis. IL-23 binding to IL23R activates the JAK/STAT3 pathway, attracting a heterodimer of JAK2 and subsequent TYK2 phosphorylation of tyrosine residues on both the JAKs and IL23R, allowing STAT3 to bind. The JAK2/TYK2 complex phosphorylates STAT3, which then translocates to the nucleus and facilitates DNA binding to target gene promoters [8,9]. To a much lesser extent, the JAK2/TYK2 complex also promotes STAT1, STAT4, and STAT5 phosphorylation [8]. In murine studies, TYK2-deficient mice have been found to be protected against disease development in experimental models of multiple sclerosis and Ps, with Th17 cells being differentiated in these mice through failed response to IL-23 stimulation [10].

Genome-wide association studies (GWASs) on Ps have made tremendous strides in understanding the genes and pathways that are involved in disease risk. Although the majority of the genetic variants implicated in disease risk are found outside protein-coding regions (exons), one notable exception is single-nucleotide polymorphisms (SNPs) within an exon of TYK2, which are consistently demonstrated to increase the risk of developing Ps.

Regarding non-coding genetic variants associated with disease risk, the vast majority of GWAS risk SNPs are enriched in gene regulation regions (enhancers) and are thought to influence the expression of target genes. Through chromatin folding, these target genes can be a large distance away from the regulatory region.

We have previously demonstrated how risk SNPs that are intronic of QTRT1 exist in active chromatin regions and make physical contact with the TYK2 gene (Supplementary Figure S1). Our hypothesis here is therefore that these risk variants do not regulate QTRT1 or, indeed, the nearby ILF3 gene, but instead increase the risk of developing Ps through the regulation of the TYK2 gene, which is already strongly implicated in the disease, not only through the associated exonic variants but also through treatments targeting this gene. Demonstrating that these risk variants do actually regulate the TYK2 gene has important consequences for understanding the genetic risk of Ps, the importance of TYK2 in disease, and how patients with an impaired genetic TYK2 response respond to therapy.

In-house capture Hi-C data indicate long-range chromatin interactions between these Ps-associated SNPs and the TYK2 gene. Assays were shown to turn this region on/off in a CD4+ T cell model (Jurkats). We then went on to characterise the consequences of this on a whole-genome expression level, with RNA-seq, to establish the TYK2 network in the developed cell model systems.

## 2. Results

### 2.1. Ps SNPs Rs892086 and rs7248205 Mapped Using UCSC Browser

**Figure 1:**
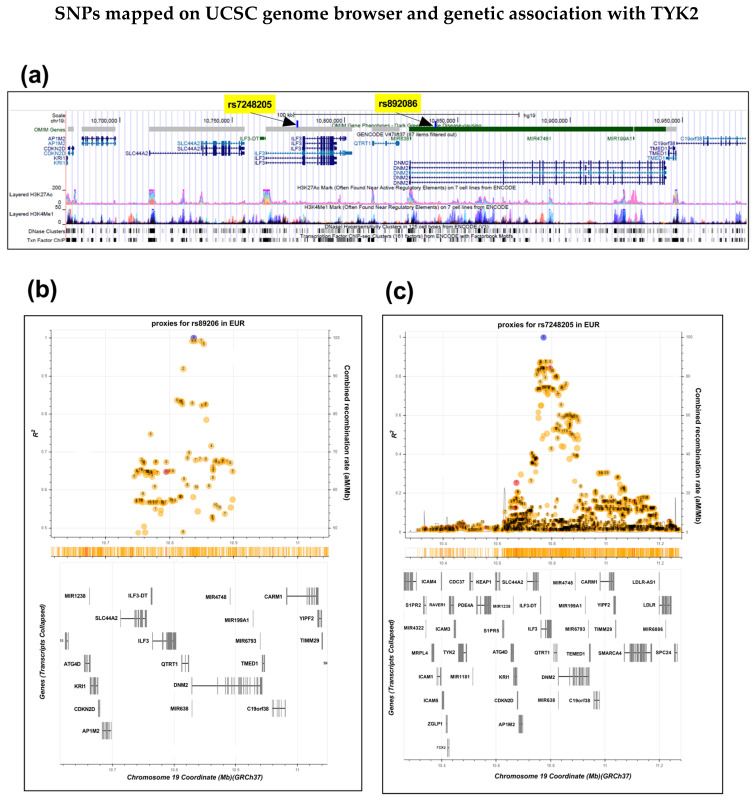
rs892086 and rs7248205 SNPs in strong LD (R^2^ > 0.8) overlapped with enhancer and promoter marks (**a**). The SNPs are eQTLs for SMARCA4, SLC44A2, and ILF3 in skin and skeletal muscle (GTEx portal). The SNPs were mapped using LD Proxy for a European population. An independent non-coding genetic association with psoriasis was identified ~400 kb upstream of TYK2, and this is shown in (**b**,**c**) for rs892086 and rs7248205 SNPs, respectively, highlighted in blue.

An independent non-coding genetic association with Ps was identified ~400 kb upstream of TYK2. An UCSC browser image showed the variants making up the credible SNP set for rs892086 (R^2^ 0.85) and another SNP, rs7248205 (R^2^ 0.8), in their genomic context [11] (Figure 1a). This provided more evidence that these variants may have the potential to influence TYK2 expression by interacting with regulatory elements involved in gene regulation. The image also shows relevant ENCODE histone and DNA accessibility data. The SNPs were mapped using LD Proxy for a European population. An independent non-coding genetic association with Ps was identified (Figure 1b,c).

### 2.2. Ps SNP and TYK2 Interaction

**Figure 2:**
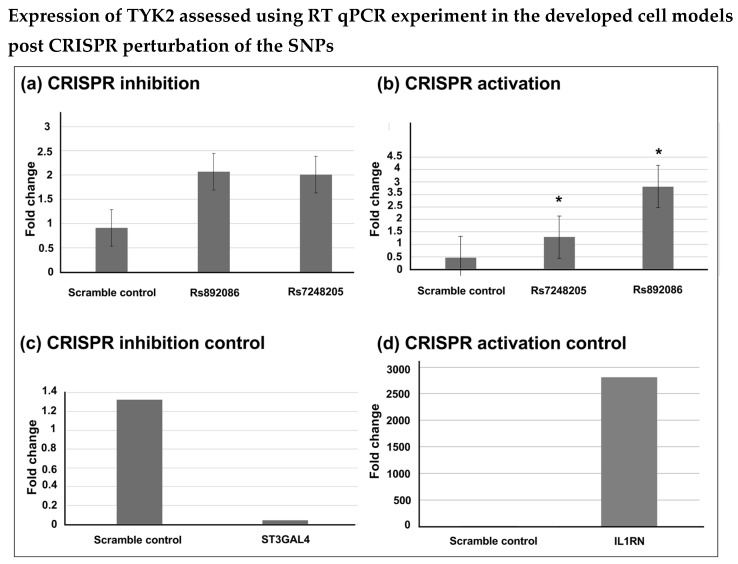
The expression of TYK2 was assessed using the RT qPCR experiment. TYK2 gene expression was measured using quantitative real-time PCR. The results from the inhibition experiment are shown in (**a**). The results from the CRISPR activation experiment are shown in (**b**), comparing the SNPs rs892086 and rs7248205 with the scramble negative control. IL1RN and ST3GAL4 were used as positive controls for the CRISPR activation and inhibition experiments, respectively, and are shown in (**c**,**d**). The expression of genes can be seen as a fold change in the graph. Triplicates were used for each test. The fold change was measured using the 2^−∆∆Ct^ method by normalising the geometric mean of the Ct values of the housekeeping genes GAPDH and YWHAZ and the variation between genes. Data were normalised against the background. Experiments were analysed using a *t* test and non-parametric Mann–Whitney test (* *p* < 0.05). Data are presented as means +/− SD and are representative of 3 independent experiments.

The Ps SNP rs892086 was selected for the development of a cell model in the Jurkat-dCas9-VP64 CD 4 T cell line. These Ps risk SNPs are intronic of ILF3 (Figure 2a). rs892086 exists within an intron of QTRT1 and undergoes a long-range interaction with TYK2. CRISPR activation and CRISPR inhibition experiments were used to develop the cell models using Jurkat-dCAS9-VP64 and Jurkat-dCAS9-KRAB cells, respectively. Two Ps SNPs, rs892086 and rs7248205, were investigated in this study. The lentivirus delivery method was used to deliver the designed gRNAs targeting the SNPs. Jurkat-dCAS9-V64 cells and Jurkat-dCAS9-KRAB cells transduced with lentivirus with gRNAs were expanded using antibiotic selection media. The expression of TYK2 was assessed using RT qPCR. No significant difference was observed between the two SNPs and the scramble control in the CRISPR interference experiment (Figure 2a). It was observed that the TYK2 expression in the Jurkat-dCAS9-VP64 CD4 T cells was enhanced post CRISPR activation of rs892086 when compared to that of the scramble control and the other SNP, rs7248205 (Figure 2b). The ST3GAL4 gene was used as a positive control for the inhibition experiment (Figure 2c), and IL1RN was used as a positive control Figure 2d).

### 2.3. RNA-Seq Identifies Differential Regulation of Genes in CRISPR Models

The effect on whole-genome expression post CRISPR activation and inhibition experiments perturbing TYK2 was assessed using RNA-seq. Jurkat-dCAS9-VP64 and Jurkat-dCAS9-KRAB CD4 T cells were used for the CRISPR activation and inhibition experiments, respectively. For the purposes of the RNA-seq experiments, Jurkat-dCAS9-VP64-TYK2 and Jurkat-dCAS9-VP64 scramble control cells were expanded in a culture in triplicate, and total RNA was used for RNA-seq. Overall, 184 genes were observed to be upregulated, and 58 genes were observed to be downregulated.

**Figure 3:**
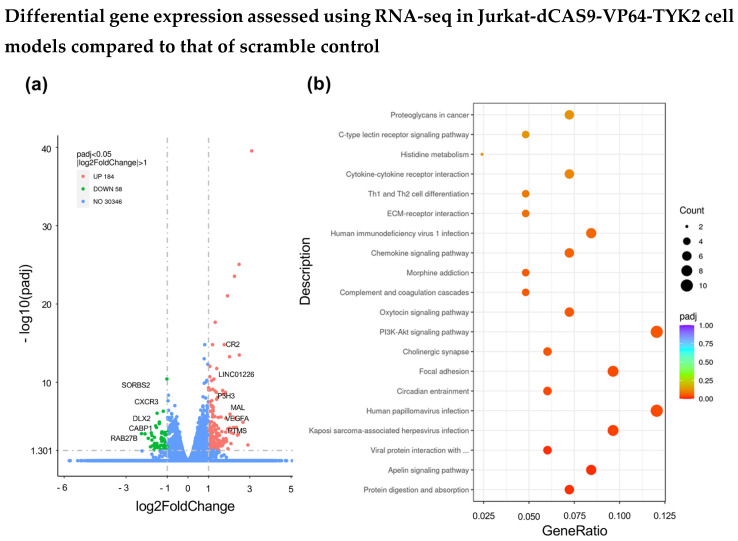

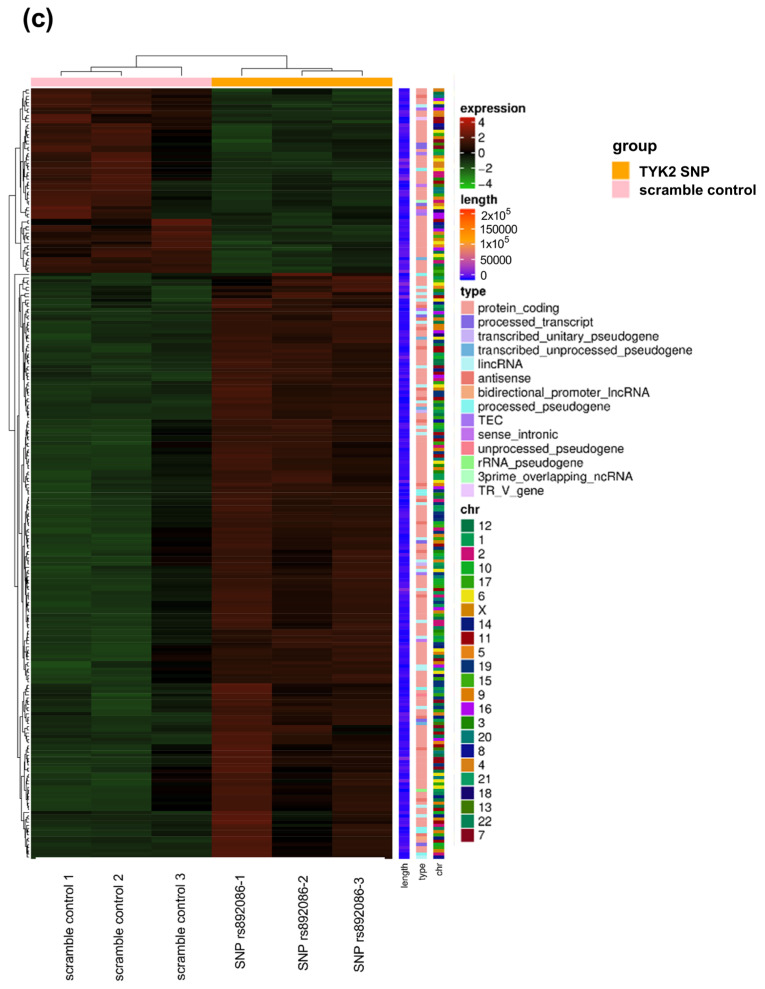
Differential gene expression comparison of Jurkat-dCAS9-VP64-TYK2 with scramble control assessed using RNA-seq experiment. Jurkat-dCAS9-VP64-TYK2 CD4+ T cells and Jurkat-dCAS9-VP64 scramble control samples were prepared using RNA extraction in triplicate. Two groups were compared to assess gene regulation. The results are shown as differential gene expression. Gene ontology results are shown for highlighted pathways in (**a**). Differential gene expression using a volcano plot is shown in (**b**), comparing Jurkat-dCAS9-VP64-TYK2 CD4+ T cells and the scramble control, followed by a heatmap comparing the two groups shown in (**c**). For both the differential gene expression and pathway analyses, results with padj < 10^−5^ are represented.

Cytokine–cytokine receptor interaction, the C-type lectin receptor signalling pathway, Th1 and Th2 cell differentiation, the cytokine signalling pathway, focal adhesion, the apelin signalling pathway, protein digestion, and the absorption pathways were observed to be enriched, as shown in Figure 3b. A heatmap comparing Jurkat-dCAS9-VP64-TYK2 and the Jurkat-dCAS9-VP64 scramble control is shown in Figure 3c, demonstrating differences in the gene sets between the triplicates from each sample set.

The top 20 differentially upregulated genes are listed in Table 1, and the top 20 differentially downregulated genes are listed in Table 2. The full list of differentially regulated genes can be found in Supplementary Table S1. We compared the full list of differentially expressed genes to genes that are implicated, through either molecular or immunology studies, in the disease or undergo physical interactions with GWAS-associated SNPs in Ps [12], and these genes are shown in Table 2.

## 3. Discussion

The TYK2 gene has been implicated in Ps through both genetic studies and its successful use as a therapeutic target. Multiple genetic studies have consistently found exonic SNPs within the TYK2 gene to increase the risk of developing Ps. Here, we wanted to assess the possibility that other Ps variants, located within an active region intronic of the QTRT1 gene around 500 kb distal from the TYK2 gene, could actually regulate the expression of this gene through our established DNA folding. By generating model systems in the Jurkat-dCAS9-VP64 and Jurkat-dCAS9-KRAB CD4+ T cell lines using CRISPR activation and inhibition experiments, we did find evidence that the active DNA region around the associated SNPrs892086 [13] could indeed influence the regulation of TYK2, measured through RT qPCR.

We went on to characterise the consequences of activating this SNP region on whole-cell expression with RNA-seq and establish the TYK2 network in these cells and the pathways impacted downstream. Recently, Shi et al. produced a list of likely causal genes in Ps based on HiChIP interactions between Ps-associated variants and their target genes [12]. We compared the differentially expressed genes generated using RNA-seq data from the Jurkat-dCAS9 activation models for rs892086 and identified key genes, such as VEGFA, C1R, ADORA1, GLUD2, NDUFB8, and FCGR2C, that are differentially regulated when the queried risk SNP, in a long-range interaction with TYK2, is perturbed.

Deucravacitinib (SOTYKTU™) is a highly selective oral TYK2 inhibitor, first approved for the treatment of Ps by the FDA, then in Japan in 2022, then for use in Europe in 2023 [14]. Tyrosine kinase inhibitors (JAK1-3) bind to the active tyrosine kinase domain, acting through competitive inhibition, whereas Deucravacitinib binds to the catalytically inactive pseudokinase regulatory domain [15]. This mechanism of action inhibits the receptor-mediated recruitment of TYK2 and prevents downstream signalling [15]. For the IL-23 signalling pathway in particular, TYK2 inhibition has a reduced risk of potential side effects compared to JAK2, as it is used by fewer cytokine receptors [9]. The differentially regulated genes identified in the developed model systems are associated with dermatological conditions, as shown in Table 2, such as psoriasis, systemic sclerosis, psoriatic arthritis, inflammatory dermatitis, and melanoma, implicating them as having a role in disease pathogenesis. The GWAS field assigns associated variants to genes and pathways based on proximity and scores patients based on whether they carry a risk variant. Here, we empirically determined an actual TYK2 psoriasis pathway, with genes in it that are implicated in psoriasis. Extending this work to estimate the magnitude of the effect of carrying these TYK2 pathway variants and scoring patients, for the first time, with an empirical genetic risk score will be a major advancement in stratifying patients in order to ascertain which biological pathways are driving the disease; will provide subgroups of patients for clinical trials and therapeutics; and will potentially offer novel drug targets for the affected pathway. In addition, an empirically annotated TYK2 pathway will also allow better targeting of other dermatological and autoimmune conditions, such as psoriatic arthritis and inflammatory dermatitis, as shown in this study, both of which may be best treated with such a therapy.

In this study, we demonstrated, for the first time, that risk variants do actually regulate the TYK2 gene and have important consequences in understanding the genetic risk of Ps, with the potential to be mapped to the Ps/TYK2 pathway.

## 4. Materials and Methods

### 4.1. Cell Culture

Jurkat E6.1 CD4+ T cells (ATCC, TIB 152) were grown in and maintained using culture media consisting of RPMI-1640 with L-glutamine and sodium bicarbonate (Sigma-Aldrich, Poole, UK) plus 10% heat-inactivated foetal bovine serum (FBS) (Gibco, Runcorn, UK), along with 1% penicillin/streptomycin (Sigma-Aldrich, Poole, UK). Jurkat-dCas9-VP64 and Jurkat-dCas9-KRAB cell lines were created in the laboratory using transfer vectors. For dCAS9-VPR, Lenti-EF1a-dCas9-VPR-puro (Addgene, Watertown, NY, USA) was used as the transfer vector, whereas for dCAS9-KRAB, pLV-CBh-dCas9-KRAB-MeCP2-T2A-Puro was used as the transfer vector, both containing the Puromycin resistance gene as a selection marker. Cells were grown in selection media with Puromycin (6 µg/mL).

### 4.2. gRNA Design and Production

Guide RNAs (gRNAs) were designed against the GRh38/hg 38 human reference genome using the CRISPOR website. A balance between off-target profiles and location and predicted 1- or 2-mismatch off-target was used for gRNA design. Those with overlapping common variations were discounted.

### 4.3. Lentivirus Production

LentiX HEK293T cells (Clontech, Chineham, UK, catalogue #632180) were cultured using DMEM (Lonza, Slough, UK) without penicillin/streptomycin and maintained at 37 °C, 5% CO_2_. Media were changed prior to transfection. The transfection mixture was prepared using Opti-MEM media (Gibco) containing a plasmid vector and virus packaging plasmids (pMD2.G, pMDLg/pRRE, 3 µg pRSV-Rev. Polyethylenimine (PEI) (Polyscience 23966-2)) with a 3:1 PEI–plasmid ratio. The mixture was incubated at RT for 10–12 min post vortexing and was added to the LentiX HEK293T cells and incubated for 72 hrs. The supernatant containing virus particles was collected, spun at 1200 RPM for 5 min at 4 °C, and filtered through a 0.45 μm cellulose acetate vacuum filter (Corning, Flintshire, UK). This supernatant was aliquoted and stored at −80 °C and used for transfection.

### 4.4. CRISPR Activation and Interference in CD4 Jurkat-dCAS9 T Cells

Jurkat-dCAS9-VP64 and Jurkat-dCAS9-KRAB were plated in 96-well round-bottom tissue culture plates (Corning) in Jurkat culture media in triplicate and cultured overnight. Cells were washed, and media were replaced with antibiotic free media. Polybrene (Sigma-Aldrich, Poole, UK, H9268) and a viral supernatant containing lentivirus carrying pLKO5.sgRNA.NeoR expressing the chosen sgRNA/scramble control were added to the cells. Media were exchanged post 24 h and incubated further for 72 h before transferring the cells into T25 flasks. Cells were cultured using selection media containing antibiotics followed by maintenance media with lower levels of antibiotics (Puromycin 3 µg/mL, Geneticin 150 µg/mL) for RNA extraction.

### 4.5. Gene Expression

RT qPCR gene expression analysis was performed to assess the expression of genes of interest using the TaqMan Gene Expression assay (Thermo Fisher Scientific, Runcorn, UK). Total RNA was extracted from the cells as described in later the Methods section under RNA extraction. cDNA was synthesised using the High-Capacity RNA to cDNA kit (Applied Biosystems, Birchwood, UK) according to the manufacturer’s protocol. Briefly, 2 µg of RNA per reaction (containing RT buffer, enzyme mix, nuclease-free water) was used. Tubes were sealed and centrifuged briefly to avoid air bubbles. The reaction was incubated for 60 min at 37 °C and stopped by heating at 95 °C for 5 min. cDNA samples were stored at −20 °C until use for RT qPCR experiments. The cDNA reaction mixture contained the TaqMan Gene Expression assay, nuclease-free water for dilution, and the TaqMan^TM^ Fast Universal PCR Master Mix (2×) No Amperase^TM^ UNG. Reactions were heated to 95 °C for 10 min, followed by 40 amplification cycles of 95 °C for 15 s and 60 °C for 1 min using the Quantstudio Flex 12K real-time PCR system (Applied Biosystems, Birchwood, UK). Triplicates were used for each test. Fold change was measured using the 2^−∆∆Ct^ method by normalising the geometric mean of the Ct values of the housekeeping genes GAPDH and YWHAZ and the variation between genes.

### 4.6. RNA Extraction and RNA Sequencing

Transformed Jurkat-dCas9-VP64-TYK2, Jurkat-dCas9-VP64 scramble control, Jurkat-dCas9-VP64-IL1RN, Jurkat-dCas9-KRAB-TYK2, and Jurkat-dCas9-KRAB-St3Gal4 T cells were cultured in maintenance media, and total RNA was extracted using the RNAeasy plus mini kit (Qiagen, Manchester, UK), as per the manufacturer’s protocol. Briefly, pelleted cells (<1 × 10^7^) were mixed with Buffer RLT Plus and vortexed for 30 s. Homogenised lysate was transferred to a gDNA Eliminator spin column placed in a 2mL collection tube. The lysates were centrifuged for 30 s at >800 g. The flowthrough was saved, and one volume of 70% ethanol was added, followed by 15 s of centrifugation at >800 g. The flowthrough was discarded. The, 700 µL of Buffer RW1 was added to the tube, followed by centrifugation for 15 s at >800 g. The flowthrough was discarded. After this, 500 µL of Buffer RPE was added to the tube, followed by centrifugation for 15 s at >800 g. This step was repeated with centrifugation for 2 min. Then, 50 µL of RNase-free water was added to the column, and this was placed in a fresh 1.5 mL collection tube, followed by centrifugation for 1 min at >800 g to elute the RNA. The RNA samples were quantified using nanodrop (Thermo Scientific NANODROP 2000 spectrophotometer, Horsham, UK), and the quality of RNA was checked using TapeStation (Agilent Technology, Santa Clara, CA, USA) prior to sequencing by Novogene Cambridge, UK.

### 4.7. Statistical Analysis

All graphs were plotted using Graphpad Prism 9. For RT qPCR, the Ct values were normalised to calculate the ΔCt values of the housekeeping genes, GAPDH and YWHAZ, and test genes. ΔΔCt was calculated for the difference between the ΔCt of the experimental and control groups. Relative expression, 2^−ΔΔCt^, was used to calculate fold change. Statistical analysis was performed with Graphpad Prism using a *t* test and non-parametric Mann–Whitney U test to compare each test group with the control group, with * *p* < 0.05, ** *p* < 0.005, and *** *p* < 0.0005. For RNA-seq data analysis, an analysis of the log2fold change results was performed by using the DESeq2 R41/EdgeR R package.

## 5. Conclusions

The activation of the region containing the Ps-implicated risk SNP rs892086 enhanced TYK2 expression over that of the Ps SNP rs7248205. We have shown here, for the first time, that the perturbation of the Ps SNP rs892086 has downstream effects on the expression of VEGFA, C1R, ADORA1, GLUD2, NDUFB8, and FCGR2C, which are also implicated in other inflammatory skin conditions through long-range interactions with associated variants. These genes could therefore form part of the TYK2 pathway, with the potential for inclusion in patient screening for the risk of developing Ps following further investigation.

## Figures and Tables

**Table 1:** A list of the top 20 upregulated and downregulated genes identified in the Jurkat-dCAS9-VP64-TYK2 CD4 T cells compared to the scramble control.

Gene Name	Padj	Differential Regulation
*CD4*	2.88 × 10^−40^	Upregulated
*LINC01226*	8.68 × 10^−26^	Upregulated
*GPR162*	2.90 × 10^−24^	Upregulated
*P3H3*	9.04 × 10^−22^	Upregulated
*MAL*	2.13 × 10^−18^	Upregulated
*CR2*	1.56 × 10^−15^	Upregulated
*PTMS*	1.56 × 10^−15^	Upregulated
*LINC01225*	3.22 × 10^−14^	Upregulated
*FRMD4A*	5.39 × 10^−14^	Upregulated
*YBX2*	9.15 × 10^−13^	Upregulated
*VEGFA*	1.72 × 10^−12^	Upregulated
*ENO2*	1.94 × 10^−11^	Upregulated
*GSC*	3.78 × 10^−11^	Upregulated
*ACY3*	5.93 × 10^−11^	Upregulated
*CCND3*	4.94 × 10^−10^	Upregulated
*AL109918.1*	7.71 × 10^−10^	Upregulated
*NEAT1*	9.41 × 10^−10^	Upregulated
*MXD3*	9.41 × 10^−10^	Upregulated
*AC136475.3*	1.09 × 10^−9^	Upregulated
*AC123912.4*	1.28 × 10^−9^	Upregulated
*CXCR3*	3.78 × 10^−11^	Downregulated
*DLX2*	4.87 × 10^−7^	Downregulated
*WWC1*	8.85 × 10^−7^	Downregulated
*MTUS1*	1.19 × 10^−5^	Downregulated
*TATDN2P2*	1.88 × 10^−5^	Downregulated
*SORBS2*	3.54 × 10^−5^	Downregulated
*ALDH1B1*	5.79 × 10^−5^	Downregulated
*CABP1*	0.000207	Downregulated
*RAB27B*	0.000235	Downregulated
*UCKL1-AS1*	0.000245	Downregulated
*KLHL29*	0.000282	Downregulated
*MAF*	0.000282	Downregulated
*MSLNL*	0.000287	Downregulated
*MIR17HG*	0.000292	Downregulated
*JAG1*	0.00031	Downregulated
*MAGEA10*	0.000354	Downregulated
*AP005131.6*	0.000373	Downregulated
*SLC49A3*	0.000505	Downregulated
*HS3ST3B1*	0.00051	Downregulated
*P2RY1*	0.000599	Downregulated

**Table 2:** Identified genes * in the current model that overlap with other skin conditions.

Dermatological Conditions	Genes Identified Using Current Model
Ps	*ADORA1*, *C1R*, *FCGR2C*, *GLUD2*, *NDUFB8*
PsA	*ADORA1*, *C1R*, *FCGR2C*, *GLUD2*, *NDUFB8*
Systemic sclerosis	*VEGFA*, *ADORA1*, *C1R*, *FCGR2C*, *GLUD2*, *NDUFB8*
Atopic dermatitis	*C1R*, *NDUFB8*
Melanoma	*ADORA1*, *C1R*

* differentially expressed genes from Table 1 and Supplementary Data were compared to those in the HiChIP database.

## Data Availability

All the data generate is available in the Supplementary Materials.

## Supplementary Materials

The following supporting information can be downloaded at: https://www.mdpi.com/article/10.3390/ijms252313229/s1.
