# Supplementary material for: Characterising a Novel Therapeutic Target for Psoriasis, TYK2, Using Functional Genomics
Source: Int J Mol Sci. 2024 Dec 9;25(23):13229. doi: 10.3390/ijms252313229 (PMC11642893; doi:10.3390/ijms252313229)
Supplement: Supplementary file 1 [file ijms-25-13229-s001.zip › ijms-3255006-supplementary.pdf]

Supplementary Materials:

Supplementary Table S1, list of differentially regulated genes identified in the Jurkat-dCAS9-VP64-TYK2 CD4 T cells compared to that of scramble control

padj	*gene_name*
2.88E-40	*CD4*
8.68E-26	*LINC01226*
2.90E-24	*GPR162*
9.04E-22	*P3H3*
2.13E-18	*MAL*
1.56E-15	*CR2*
1.56E-15	*PTMS*
3.22E-14	*LINC01225*
5.39E-14	*FRMD4A*
9.15E-13	*YBX2*
1.72E-12	*VEGFA*
1.94E-11	*ENO2*
3.78E-11	*CXCR3*
3.78E-11	*GSC*
5.93E-11	*ACY3*
4.94E-10	*CCND3*
7.71E-10	*AL109918.1*
9.41E-10	*NEAT1*
9.41E-10	*MXD3*
1.09E-09	*AC136475.3*
1.28E-09	*AC123912.4*
1.92E-09	*KCNN4*
1.92E-09	*RAB3D*
3.30E-09	*BMF*
8.66E-09	*GNB3*
1.44E-08	*TNNT1*
2.21E-08	*C10orf25*
3.07E-08	*TPM2*
8.36E-08	*SAT2*
9.65E-08	*ZNF629*
1.14E-07	*KANK2*
1.36E-07	*PRR33*
2.71E-07	*PPFIA4*
4.80E-07	*BHLHE40*
4.87E-07	*DLX2*
4.89E-07	*EBF4*
4.89E-07	*ADGRG1*
8.85E-07	*WWC1*
1.16E-06	*TMIGD2*
1.30E-06	*RASIP1*
2.35E-06	*LRRC38*

2.58E-06	*AC004585.1*
2.71E-06	*ADIRF-AS1*
2.98E-06	*RAB33A*
3.75E-06	*AL121612.2*
4.04E-06	*AL031118.1*
1.06E-05	*AC008105.3*
1.19E-05	*MTUS1*
1.24E-05	*SDK2*
1.25E-05	*NALT1*
1.75E-05	*LINC00051*
1.88E-05	*TATDN2P2*
2.28E-05	*RAB44*
3.20E-05	*CCR8*
3.45E-05	*HNRNPA1P21*
3.54E-05	*SORBS2*
4.20E-05	*RGS9*
5.57E-05	*COL6A2*
5.79E-05	*AC092490.1*
5.79E-05	*ALDH1B1*
5.80E-05	*RNLS*
6.25E-05	*EFHD1*
6.88E-05	*SLC14A1*
6.91E-05	*AL021368.2*
7.64E-05	*ALDH3B2*
8.55E-05	*LINC00173*
0.000105	*C2CD4C*
0.000109	*GPC3*
0.00011	*LINC00461*
0.000114	*C1RL*
0.000116	*ANKRD20A19P*
0.000123	*OLMALINC*
0.00016	*CD72*
0.000179	*ZNF436-AS1*
0.000194	*PNCK*
0.000207	*CABP1*
0.000211	*AC136475.2*
0.00023	*CYP4F2*
0.000235	*RAB27B*
0.000245	*UCKL1-AS1*
0.000254	*AC005336.3*
0.000282	*KLHL29*
0.000282	*EFEMP2*
0.000282	*MAF*
0.000287	*MSLNL*
0.000292	*MIR17HG*
0.00031	*JAG1*
0.000331	*AC005336.2*
0.000345	*NFATC4*

0.000353	*LINC00894*
0.000354	*MAGEA10*
0.000373	*AP005131.6*
0.000382	*PLAUR*
0.000421	*PIK3IP1*
0.000427	*TRPM5*
0.000505	*SLC49A3*
0.00051	*HS3ST3B1*
0.000547	*L1CAM*
0.000554	*AC104794.2*
0.000599	*P2RY1*
0.000673	*STPG3*
0.000717	*EFNA3*
0.000773	*AC138904.1*
0.000778	*IFI35*
0.000857	*AC010616.1*
0.000943	*KEL*
0.001038	*TLL1*
0.001054	*TMEM200A*
0.001246	*COL9A2*
0.0014	*HRAT92*
0.001416	*GRIP1*
0.001428	*CELF5*
0.001431	*LINC00892*
0.001529	*TNFAIP2*
0.00158	*IPCEF1*
0.001639	*PIK3R6*
0.001685	*ENPP1*
0.001776	*Sep-04*
0.001894	*HAGLR*
0.001947	*ARHGEF4*
0.002216	*C9*
0.002321	*SOX5*
0.002325	*AC000078.1*
0.00237	*H1F0*
0.002558	*AC021054.1*
0.002705	*TMC7*
0.002819	*RAMP2*
0.002837	*PPP1R32*
0.002928	*HLA-F*
0.002936	*CCR7*
0.003006	*AC008115.3*
0.003149	*AC097534.2*
0.003305	*MYL4*
0.003408	*AC027307.3*
0.003544	*TTLL9*
0.00356	*ALPK1*
0.003623	*C16orf74*

0.003788	*AL513497.1*
0.003835	*SERPINH1P1*
0.003999	*GCSAM*
0.004245	*NHLRC4*
0.004554	*TPO*
0.004711	*C11orf21*
0.004917	*CDHR1*
0.005241	*SYNM*
0.005332	*AC123912.2*
0.005642	*LSP1*
0.005827	*CFAP73*
0.006162	*ZNF365*
0.006243	*CACNA1D*
0.006281	*ARHGEF40*
0.006556	*TEAD2*
0.006969	*AC110285.6*
0.007242	*GNG7*
0.007358	*GRID2IP*
0.007776	*PDE10A*
0.009084	*C1QL4*
0.009084	*DENND6B*
0.009097	*AC233968.1*
0.009284	*RYR3*
0.009528	*JAKMIP3*
0.009857	*ADM*
0.009857	*AC007262.2*
0.009857	*TMEM74B*
0.01006	*HOXC11*
0.010248	*SSPO*
0.010425	*AC116565.1*
0.010515	*LRIT3*
0.010757	*MYOM1*
0.010807	*PRSS2*
0.011275	*IL24*
0.011444	*EGR3*
0.011593	*NLRP6*
0.012354	*STPG3-AS1*
0.012623	*C11orf74*
0.012961	*CLDN5*
0.012997	*SWAP70*
0.013182	*ERVH48-1*
0.01352	*SMIM17*
0.01352	*RPL29P11*
0.013742	*PLTP*
0.013818	*CCDC146*
0.01457	*AC019257.1*
0.014589	*AC012358.2*
0.014687	*NXF3*

0.014687	*RNA5SP18*
0.014687	*AC135048.4*
0.015001	*MME*
0.016021	*AC108025.2*
0.016021	*TRAF1*
0.016348	*ZNF230*
0.016388	*LINC00029*
0.016903	*MKLN1-AS*
0.016936	*AKR1C1*
0.016963	*AC120114.3*
0.017197	*ZNF451-AS1*
0.01722	*COL9A3*
0.017259	*PYY2*
0.01784	*AL137002.2*
0.018025	*HCG27*
0.018151	*PROM2*
0.020159	*ADORA1*
0.021057	*RASA3-IT1*
0.021231	*ZBTB16*
0.021611	*FLNC*
0.021752	*BX571818.1*
0.022137	*GRAMD2A*
0.02241	*SLC30A3*
0.022823	*SLC43A2*
0.023028	*ARPP21-AS1*
0.023531	*TNXB*
0.023941	*AC103563.7*
0.02586	*DANCR*
0.027295	*CDYL2*
0.027509	*AC011767.1*
0.028611	*AL365273.1*
0.029553	*MIR210HG*
0.030201	*TSPAN11*
0.030288	*FOS*
0.030301	*AC135782.2*
0.031201	*AC099673.1*
0.032524	*WNT10B*
0.033102	*LINC02384*
0.035621	*SPINK2*
0.036426	*JAKMIP2*
0.038978	*CNKSR3*
0.04018	*C11orf94*
0.041045	*PAX6*
0.041172	*FKBP10*
0.041914	*ISM1*
0.042878	*TRGV1*
0.042878	*C1R*
0.046719	*MYLK4*

0.046981	*HBQ1*
0.046981	*MSANTD1*
0.046981	*AC096631.2*
0.048643	*AL163932.1*
0.048668	*CILP2*
0.048799	*TNF*
0.049344	*CCM2L*
0.049344	*MARCKS*
0.049406	*PYHIN1*

Upregulated

**padj**	***gene_name***
2.88E-40	*CD4*
8.68E-26	*LINC01226*
2.90E-24	*GPR162*
9.04E-22	*P3H3*
2.13E-18	*MAL*
1.56E-15	*CR2*
1.56E-15	*PTMS*
3.22E-14	*LINC01225*
5.39E-14	*FRMD4A*
9.15E-13	*YBX2*
1.72E-12	*VEGFA*
1.94E-11	*ENO2*
3.78E-11	*GSC*
5.93E-11	*ACY3*
4.94E-10	*CCND3*
7.71E-10	*AL109918.1*
9.41E-10	*NEAT1*
9.41E-10	*MXD3*
1.09E-09	*AC136475.3*
1.28E-09	*AC123912.4*
1.92E-09	*KCNN4*
1.92E-09	*RAB3D*
3.30E-09	*BMF*
8.66E-09	*GNB3*
1.44E-08	*TNNT1*
2.21E-08	*C10orf25*
3.07E-08	*TPM2*
8.36E-08	*SAT2*
9.65E-08	*ZNF629*
1.14E-07	*KANK2*
1.36E-07	*PRR33*
2.71E-07	*PPFIA4*
4.80E-07	*BHLHE40*
4.89E-07	*EBF4*
4.89E-07	*ADGRG1*

1.16E-06	*TMIGD2*
1.30E-06	*RASIP1*
2.35E-06	*LRRC38*
2.58E-06	*AC004585.1*
2.71E-06	*ADIRF-AS1*
2.98E-06	*RAB33A*
3.75E-06	*AL121612.2*
4.04E-06	*AL031118.1*
1.06E-05	*AC008105.3*
1.24E-05	*SDK2*
1.25E-05	*NALT1*
1.75E-05	*LINC00051*
2.28E-05	*RAB44*
3.20E-05	*CCR8*
3.45E-05	*HNRNPA1P21*
4.20E-05	*RGS9*
5.57E-05	*COL6A2*
5.79E-05	*AC092490.1*
5.80E-05	*RNLS*
6.25E-05	*EFHD1*
6.88E-05	*SLC14A1*
6.91E-05	*AL021368.2*
7.64E-05	*ALDH3B2*
8.55E-05	*LINC00173*
0.000105	*C2CD4C*
0.000109	*GPC3*
0.00011	*LINC00461*
0.000114	*C1RL*
0.000116	*ANKRD20A19P*
0.000123	*OLMALINC*
0.00016	*CD72*
0.000179	*ZNF436-AS1*
0.000194	*PNCK*
0.000211	*AC136475.2*
0.00023	*CYP4F2*
0.000254	*AC005336.3*
0.000282	*EFEMP2*
0.000331	*AC005336.2*
0.000345	*NFATC4*
0.000353	*LINC00894*
0.000382	*PLAUR*
0.000421	*PIK3IP1*
0.000427	*TRPM5*
0.000547	*L1CAM*
0.000554	*AC104794.2*
0.000673	*STPG3*
0.000717	*EFNA3*
0.000773	*AC138904.1*

0.000778	*IFI35*
0.000857	*AC010616.1*
0.000943	*KEL*
0.001246	*COL9A2*
0.0014	*HRAT92*
0.001428	*CELF5*
0.001431	*LINC00892*
0.001529	*TNFAIP2*
0.001639	*PIK3R6*
0.001776	*Sep-04*
0.001894	*HAGLR*
0.002325	*AC000078.1*
0.00237	*H1F0*
0.002558	*AC021054.1*
0.002705	*TMC7*
0.002819	*RAMP2*
0.002837	*PPP1R32*
0.002928	*HLA-F*
0.002936	*CCR7*
0.003006	*AC008115.3*
0.003149	*AC097534.2*
0.003305	*MYL4*
0.003544	*TTLL9*
0.00356	*ALPK1*
0.003623	*C16orf74*
0.003835	*SERPINH1P1*
0.003999	*GCSAM*
0.004245	*NHLRC4*
0.004554	*TPO*
0.004711	*C11orf21*
0.004917	*CDHR1*
0.005241	*SYNM*
0.005332	*AC123912.2*
0.005642	*LSP1*
0.005827	*CFAP73*
0.006281	*ARHGEF40*
0.006556	*TEAD2*
0.006969	*AC110285.6*
0.007242	*GNG7*
0.007358	*GRID2IP*
0.007776	*PDE10A*
0.009084	*C1QL4*
0.009084	*DENND6B*
0.009097	*AC233968.1*
0.009528	*JAKMIP3*
0.009857	*ADM*
0.009857	*AC007262.2*
0.009857	*TMEM74B*

0.01006	*HOXC11*
0.010248	*SSPO*
0.010425	*AC116565.1*
0.010757	*MYOM1*
0.011275	*IL24*
0.011593	*NLRP6*
0.012354	*STPG3-AS1*
0.012961	*CLDN5*
0.012997	*SWAP70*
0.013182	*ERVH48-1*
0.01352	*SMIM17*
0.01352	*RPL29P11*
0.013742	*PLTP*
0.013818	*CCDC146*
0.01457	*AC019257.1*
0.014589	*AC012358.2*
0.014687	*NXF3*
0.014687	*RNA5SP18*
0.015001	*MME*
0.016021	*TRAF1*
0.016348	*ZNF230*
0.016388	*LINC00029*
0.016936	*AKR1C1*
0.01722	*COL9A3*
0.01784	*AL137002.2*
0.018025	*HCG27*
0.018151	*PROM2*
0.020159	*ADORA1*
0.021611	*FLNC*
0.021752	*BX571818.1*
0.02241	*SLC30A3*
0.023028	*ARPP21-AS1*
0.023531	*TNXB*
0.023941	*AC103563.7*
0.027509	*AC011767.1*
0.028611	*AL365273.1*
0.029553	*MIR210HG*
0.030201	*TSPAN11*
0.030288	*FOS*
0.030301	*AC135782.2*
0.031201	*AC099673.1*
0.032524	*WNT10B*
0.033102	*LINC02384*
0.035621	*SPINK2*
0.041045	*PAX6*
0.041172	*FKBP10*
0.042878	*C1R*
0.046981	*AC096631.2*

0.048643	*AL163932.1*
0.048799	*TNF*
0.049344	*CCM2L*
0.049344	*MARCKS*
0.049406	*PYHIN1*

Downregulated

**padj**	***gene_name***
3.78E-11	*CXCR3*
4.87E-07	*DLX2*
8.85E-07	*WWC1*
1.19E-05	*MTUS1*
1.88E-05	*TATDN2P2*
3.54E-05	*SORBS2*
5.79E-05	*ALDH1B1*
0.000207	*CABP1*
0.000235	*RAB27B*
0.000245	*UCKL1-AS1*
0.000282	*KLHL29*
0.000282	*MAF*
0.000287	*MSLNL*
0.000292	*MIR17HG*
0.00031	*JAG1*
0.000354	*MAGEA10*
0.000373	*AP005131.6*
0.000505	*SLC49A3*
0.00051	*HS3ST3B1*
0.000599	*P2RY1*
0.001038	*TLL1*
0.001054	*TMEM200A*
0.001416	*GRIP1*
0.00158	*IPCEF1*
0.001685	*ENPP1*
0.001947	*ARHGEF4*
0.002216	*C9*
0.002321	*SOX5*
0.003408	*AC027307.3*
0.003788	*AL513497.1*
0.006162	*ZNF365*
0.006243	*CACNA1D*
0.009284	*RYR3*
0.010515	*LRIT3*
0.010807	*PRSS2*
0.011444	*EGR3*
0.012623	*C11orf74*
0.014687	*AC135048.4*
0.016021	*AC108025.2*
0.016903	*MKLN1-AS*

0.016963	*AC120114.3*
0.017197	*ZNF451-AS1*
0.017259	*PYY2*
0.021057	*RASA3-IT1*
0.021231	*ZBTB16*
0.022137	*GRAMD2A*
0.022823	*SLC43A2*
0.02586	*DANCR*
0.027295	*CDYL2*
0.036426	*JAKMIP2*
0.038978	*CNKSR3*
0.04018	*C11orf94*
0.041914	*ISM1*
0.042878	*TRGV1*
0.046719	*MYLK4*
0.046981	*HBQ1*
0.046981	*MSANTD1*
0.048668	*CILP2*

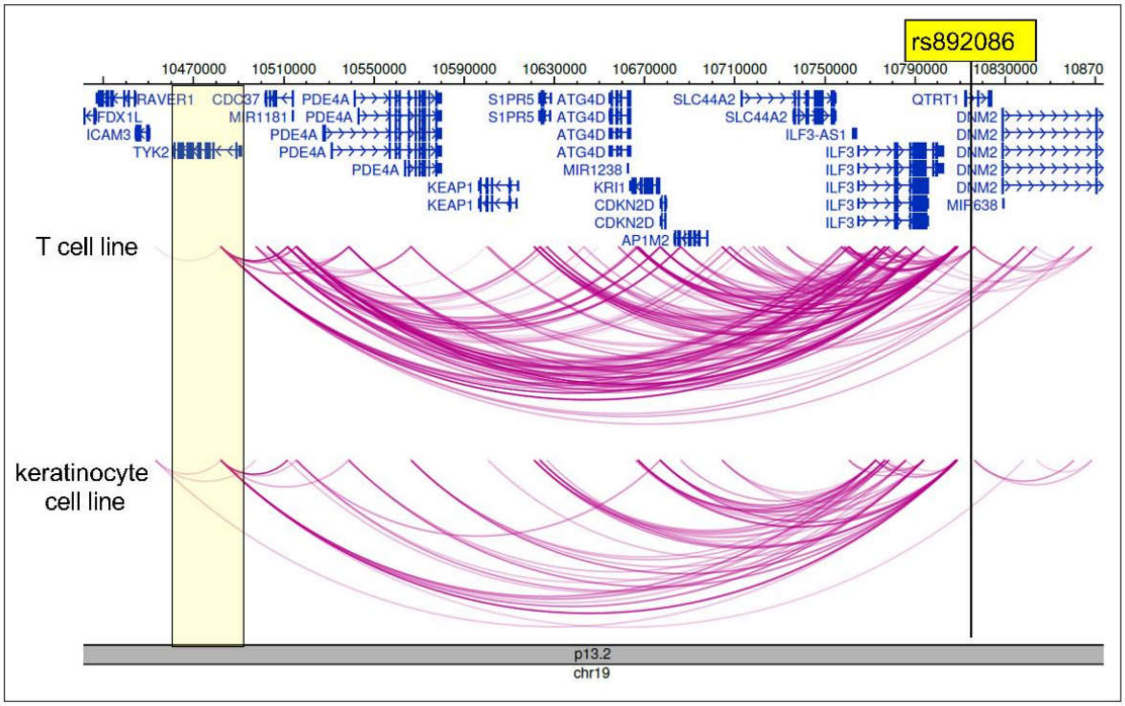

Supplementary Figure S1. Long range chromatin interaction between SNP and TYK2. Our inhouse Capture Hi-C data indicates log range physical chromatin interaction between the Ps risk SNP, rs892086, which I present at distal regulator of proriasis risk gene TYK2. The image was created using WashU Epigenome browser.
